# Microscopic Invasion of Nerve Is Associated With Aggressive Behaviors in Pancreatic Neuroendocrine Tumors

**DOI:** 10.3389/fonc.2021.630316

**Published:** 2021-02-25

**Authors:** Hao Zhou, Yajie Wang, Chuangen Guo, Xiaoshuang Li, Wenjing Cui, Zhongqiu Wang, Xiao Chen

**Affiliations:** ^1^ Department of Radiology, Affiliated Hospital of Nanjing University of Chinese Medicine, Nanjing, China; ^2^ Department of Radiology, The First Affiliated Hospital, School of Medical College, Zhejiang University, Hangzhou, China; ^3^ Department of Radiology, The Second Affiliated Hospital of Nanjing Medical University, Nanjing, China

**Keywords:** pancreatic neuroendocrine tumor, nerve invasion, aggressive behavior, clinicopathological features, predict

## Abstract

**Objectives:**

The role of neural invasion has been reported in cancers. Few studies also showed that neural invasion was related to survival rate in patients with pancreatic neuroendocrine tumor (PNET). The aim of this study is to explore the association between neural invasion and aggressive behaviors in PNET.

**Methods:**

After excluding those patients with biopsy and with missing histological data, a total 197 patients with PNET who underwent surgery were retrospectively analyzed. The demographic data and histological data were obtained. Aggressive behavior was defined based on extra-pancreatic extension including vascular invasion, organ invasion and lymph node metastases. Logistic regression analyses were used to identify risk factor for aggressive behavior. Receiver operating characteristic (ROC) curves were performed to show the performance of nomograms in evaluating aggressive behavior of PNET.

**Results:**

The prevalence of neural invasion in the cohort was 10.1% (n = 20). The prevalence of lymph node metastasis, organ invasion, and vascular invasion in PNET patients with neural invasion was higher than those in patients without neural invasion (p < 0.05). Neural invasion was more common in grade 3 (G3) tumors than G1/G2 (p < 0.01). Tumor size, tumor grade, and neural invasion were independent associated factors of aggressive behavior (p < 0.05) after adjusting for possible cofounders in total tumors and G1/G2 tumors. Two nomograms were developed to predict the aggressive behavior. The area under the ROC curve was 0.84 (95% confidence interval (CI): 0.77–0.90) for total population and was 0.84 (95% CI: 0.78–0.89) for patients with G1/G2 PNET respectively.

**Conclusions:**

Neural invasion is associated with aggressive behavior in PNET. Nomograms based on tumor size, grade and neural invasion show acceptable performances in predicting aggressive behavior in PNET.

## Introduction

Pancreatic neuroendocrine tumors (PNETs) constitute a group of rare tumors which arise from neuroendocrine cells of the pancreas (1). The current detection rate of incidental PNET during abdominal physical examination is increasing and the incidence is about 1–2% of all pancreatic tumors (2). All PNETs are regarded as potentially malignant tumors and may have several aggressive behaviors including vascular invasion, organ invasion and distant metastasis. Previous studies have shown that tumor morphology and diabetes may be associated with tumor aggressive behaviors (3, 4). Unfortunately, the role of neural invasion in PNET has not been systematically studied.

Neural invasion has been recognized as an important factor in the spread of carcinoma and as an independent prognostic factor (5). The presence of neural invasion has been reported in pancreatic cancer, prostate cancer, and colorectal cancer (6). Moreover, it has been shown that neural invasion is associated with poor prognosis and short survival in pancreatic cancer (7). Recently, several studies also showed that the neural invasion occurs in PNET. Fan et al. (4) demonstrated that neural invasion was associated with PNET-related diabetes. Han et al. (8) and Tsutsumi et al. (9) both indicated that neural invasion was also related to low survival rate in PNET patient. Some studies have shown the associated factors of aggressive behaviors in PNET (10, 11). Association between neural invasion and inguinal lymph node metastases has been reported in several types of cancer, such as penile cancer (12), gastric cancer (13), and colorectal cancer (14). However, the association between neural invasion and aggressive behavior in PNET, such as vascular/organ invasion and lymph node metastases, has not been clarified.

In this study, we provide the first demonstration of relationships between neural invasion and other aggressive behaviors in patients with PNET through a retrospective study including 197 patients.

## Materials and Methods

### Patients

The approval for this retrospective analysis was obtained from the institutional review board of the First Affiliated Hospital, Zhejiang University School of Medicine. After excluding those patients with biopsy, a total of 242 patients with PNET who underwent surgery during 2011–2019 were included in this study. Those patients with missing histological data, such as tumor grade (n = 2), lymph node status (n = 4), organ invasion and vascular invasion (n = 5) were excluded. Those patients with no information of neural invasion were also excluded (n = 34). Finally, a total of 197 patients were included.

### Histology of PNET and Definition of Aggressive Behavior

PNET grade was evaluated based on the mitotic count and the Ki-67 index (15, 16) according to the criteria of the WHO 2017 classification. PNET was divided into PNET grade 1 (PNET G1), mitotic count of <2/10 hpf or <3% Ki-67 index; PNET grade 2 (PNET G2), mitotic count of 2–20/10 hpf or 3–20% Ki-67 index; and grade 3 (PNET G3), mitotic count of >20/10 HPF or >20% Ki-67 index. Ki-67 index and mitotic count were evaluated by counting at least total 500 cells in “hot spots”. PNET G3 which included well differentiated NET G3 and neuroendocrine carcinoma (PNEC). Macroinvasion and microinvasion of adjacent organs, microvascular invasion, microlymphatic invasion and microscopic invasion of nerve were also recorded. Aggressive behavior was defined based on extra-pancreatic extension (EPE) (11), including vascular invasion, organ invasion, and lymph node metastases. Distant metastasis was also an important marker of aggressive behavior. However, those patients were not included in our study.

### Statistical Analysis

First, we divided PNET patients into two groups according to presence or absence of neural invasion. The continuous data were shown as means ± SD and qualitative data was shown as number (percentage). Clinicopathological variables including patient’s age, sex, tumor sizes, Ki67 index, tumor grade (G1 *vs* G2 *vs* G3), tumor location (Head–neck *vs* Body *vs* Tail), metastasis of lymph node, organ invasion and vascular invasion were compared subsequently by Independent-sample T test (continuous data) or Chi-square test or Fisher’s exact test (qualitative data). Univariable and multivariable logistic regression analyses were used to identify the risk factor for aggressive behavior in total population and in patients with G1/G2 PNET or G3 PNET. The results were additionally adjusted with diabetes. We also developed nomograms to predict the aggressive behavior. Calibration curve was calculated by Bootstrap self-sampling and internal verification. Receiver operating characteristic (ROC) curves were used to show the performance of nomograms in evaluating aggressive behavior of PNET. P values less than 0.05 were considered as statistically significant.

## Results

### Patient Demographics and Clinicopathological Features of PNET

A total of 197 patients were included in this study, and the clinical data are shown in **Table 1**. There were 20 patients with microscopic neural invasion. No significant differences were found in patient’s age (P = 0.59), sex (p = 0.25), tumor size (p = 0.84), tumor location (p > 0.05) and lymph node metastasis (p = 0.08) between patients with and without neural invasion. Tumor with neural invasion had a higher Ki67 index than those without neural invasion (p < 0.01). Neural invasion was more common in patients with G3 tumors than those with G1/G2 tumors (p < 0.01). Furthermore, organ invasion and vascular invasion were more common in tumor with neural invasion than in that with none (p = 0.038 and p < 0.01).

**Table 1:** Characteristic of patients based on nerve invasion.

Variables	Neural invasion (n = 20)	Non neural invasion (n = 177)	p
Age	57.30 ± 10.99	55.80 ± 11.7	0.59
Sex(Female/Male)	7/13	86/91	0.25
Length (cm)	3.48 ± 1.66	3.37 ± 2.34	0.84
Ki67	32.90 ± 31.67	13.08 ± 21.31	<0.01
Grade (1/2/3) Grade(G1+G2 *vs* G3)	2/7/11 9/11	64/78/35 142/35	0.08 <0.01
Location			0.23
Head-neck	9 (45.0%)	84 (42.4%)	
Body	10 (50.0%)	56 (26.7%)	
Location			
Head–neck–body	19 (95.0%)	140 (79.1%)	0.052
Tail	1 (5.0%)	37 (20.9%)	
Lymph node metastasis	4 (20.0%)	14 (7.9%)	0.08
Organ invasion	8 (40.0%)	35 (19.8%)	0.038
Vascular invasion	11(55.0%)	16 (9.04%)	<0.01

### Relationship Between Aggressive Behavior and Clinicopathological Features

Univariable and multivariable logistic regression analyses were used to identify the associated factor of aggressive behavior of PNET in the total population (**Table 2**) and G1/G2 patients (**Table 3**). Univariable analysis showed that tumor sizes, tumor grade, neural invasion, and lymph node metastasis were the risk factors for aggressive behavior in the total population (**Table 2**). Multivariable logistic regression analysis further identified that tumor sizes, tumor grade, and neural invasion were associated factors of aggressive behavior. The odds ratio (OR) was 1.26 (95% confidence interval (CI): 1.03–1.52) for tumor sizes and 8.55 (95% CI: 2.30–31.86) for nerve invasion. G2 and G3 PNET also had a higher risk than G1 tumor. The ORs were 4.98 (95% CI: 1.48–16.81) and 11.31 (95% CI: 3.02–42.43), respectively.

**Table 2:** Logistic regression analysis in total population.

Variables	Aggressive behavior		
	Univariable	Multivariable	Model2
	OR (95% CI)	OR (95% CI)	OR (95% CI)
Age	1.02(0.99–1.05)	1.01(0.98–1.04)	1.02(0.97–1.04)
Sex (male *vs* female)	1.57(0.85–2.93)	1.26(0.57–2.74)	1.27(0.58–2.78)
Size (cm)	1.40(1.19–1.66)	1.26(1.04–1.52)	1.26(1.03–1.52)
Grade			
G1	1	1	1
G2	7.90(2.60–24.01)	4.88 (1.46–16.30)	4.98(1.48–16.81)
G3	24.11(7.47–77.82)	11.22(3.01–41.81)	11.31(3.02–42.43)
Location			
Head–neck	1	1	1
Body	1.70(0.86–3.35)	1.45(0.62–3.37)	1.45(0.62–3.37)
Tail	0.85(0.35–2.05)	0.81(0.29–2.28)	0.82(0.29–2.33)
Nerve invasion (yes *vs* no)	12.00(3.80–37.85)	9.12(2.48–33.59)	8.55(2.30–31.86)

Model 2 was additionally adjusted with diabetes.

**Table 3:** Logistic regression analysis in patients with G1/G2 PNET (n = 147).

Variables	Aggressive behavior		
	Univariable	Multivariable	Model2
	OR (95%CI)	OR (95%CI)	OR (95%CI)
Age	1.01(0.98–1.05)	1.01(0.97–1.05)	1.00(0.96–1.05)
Sex (male vs female)	1.00(0.45–2.21)	1.22(0.48–3.08)	1.26(0.50–3.20)
Size (cm)	1.51(1.21–1.87)	1.42(1.10–1.82)	1.40(1.09–1.80)
Grade			
G1	1	1	1
G2	7.90(2.60–24.01)	3.96(1.18–13.28)	4.17(1.21–14.35)
Location			
Head–neck	1	1	1
Body	2.12(0.88–5.13)	1.38(0.50–3.81)	1.36(0.49–3.77)
Tail	1.11(0.35–3.54)	0.93(0.26–3.31)	0.95(0.26–3.47)
Nerve invasion (yes *vs* no)	5.34(1.34–21.26)	6.01(1.25–29.01)	4.86(1.01–23.59)

Model 2 was additionally adjusted with diabetes.

PNETs, pancreatic neuroendocrine tumors.

Subsequently, we identified the associated factors of aggressive behavior in patients with G1/G2 PNET (**Table 3**) and G3 PNET (**Table 4**). Multivariable logistic regression analysis showed that tumor sizes, tumor grade, and neural invasion were associated factors of aggressive behavior in G1/G2 PNET. The odds ratio (OR) was 1.40 (95% CI: 1.09–1.80) for tumor sizes, 4.86 (95% CI: 1.01–23.59) for neural invasion and 4.17 (95% CI: 1.21–14.35) for G2 tumor. Neural invasion was also an independent associated factor for aggressive behavior in G3 PNET (OR = 24.78, 95% CI:1.68–365.81).

**Table 4:** Logistic regression analysis in evaluating aggressive behavior in patients with G3 PNET (n = 50).

Variables	Model 1	Model 2
	OR (95% CI)	OR (95% CI)
Age	1.00(0.93–1.07)	1.00(0.93–1.07)
Sex (male vs female)	4.56(0.78–26.51)	3.71(0.61–22.54)
Size (cm)	1.01(0.81–1.26)	0.99(0.79–1.24)
Location		
Head–neck	1	1
Body	1.42(0.28–7.16)	1.18(0.22–6.41)
Tail	0.47(0.07–3.04)	0.40(0.06–2.70)
Nerve invasion (yes vs no)	19.68(1.88–205.67)	24.78(1.68–365.81)

Model 2 was additionally adjusted with diabetes.

PNET, pancreatic neuroendocrine tumors.

### Nomogram to Predict Aggressive Behavior

**Figure 1:**
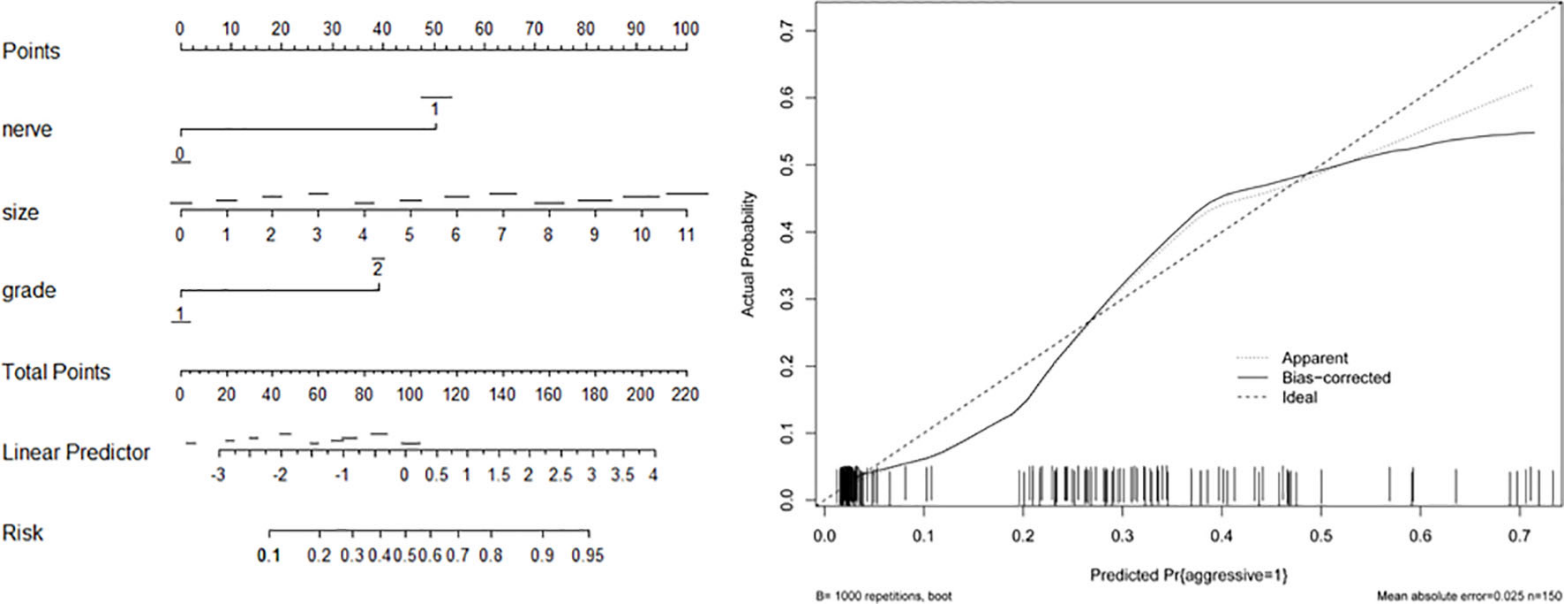
Nomogram (left) and calibration curve (right) of aggressive behavior of pancreatic neuroendocrine tumors (PNETs) in total patients. Neural invasion (nerve), tumor size, and tumor grade were included in the gram.

**Figure 2:**
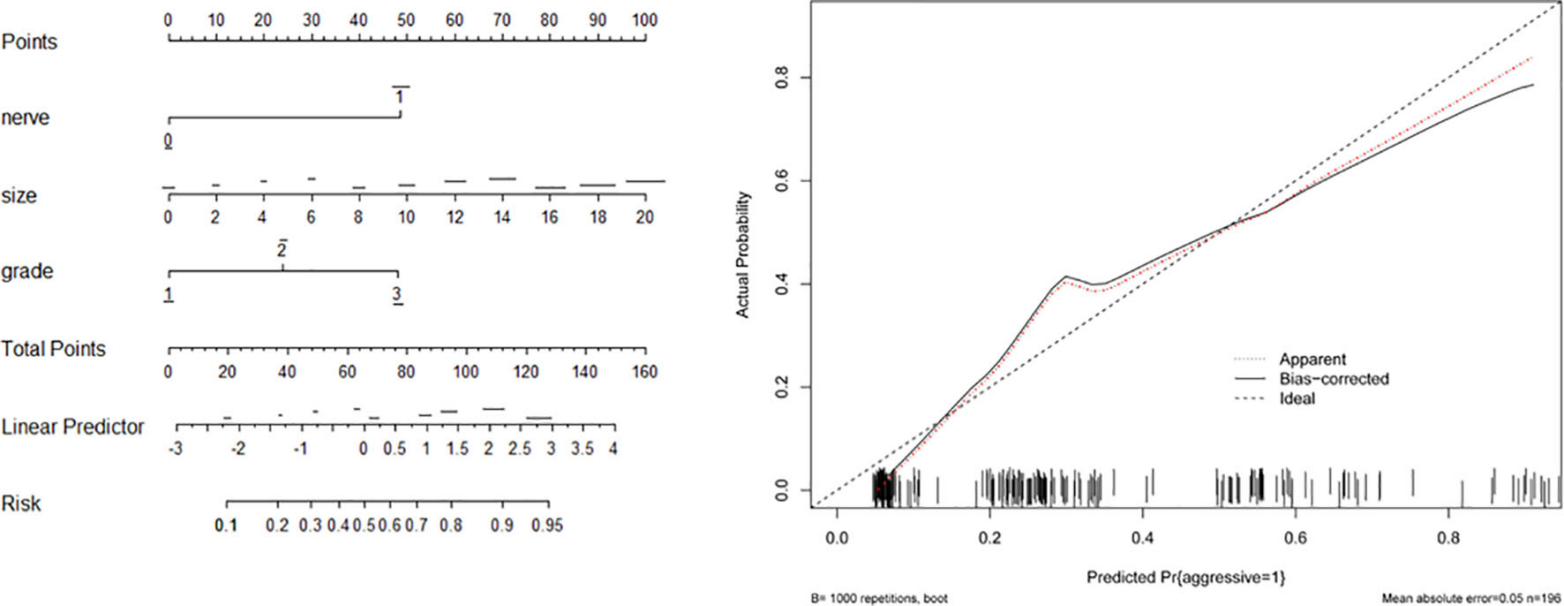
Nomogram (left) and calibration curve (right) of aggressive behavior of pancreatic neuroendocrine tumors (PNET) in G1/G2 patients. Neural invasion (nerve), tumor size and tumor grade were included in the gram.

Based on the associated factors obtained from logistic regression analyses, we developed two nomograms to predict the aggressive behavior of PNET. For total patients (**Figure 1**) and G1/G2 population (**Figure 2**), tumor size, grade, and neural invasion were included in nomogram. A score was calculated based on the three factors on the point scale. Then, we obtained a sum of each score (total points). Based on the total points, we obtained the risk on the probability scale. For example, in total population, the point was 25 for G2 tumor, and 20 for tumor with a size of 4.0 cm. The total point was 46, and the risk of aggressive behavior was 0.28. If there were neural invasion, the point for neural invasion was 49. The total point was increased to 95 and the risk of aggressive behavior was 0.80. The calibration plots of the nomograms both showed acceptable performance.

### ROC Analysis

**Figure 3:**
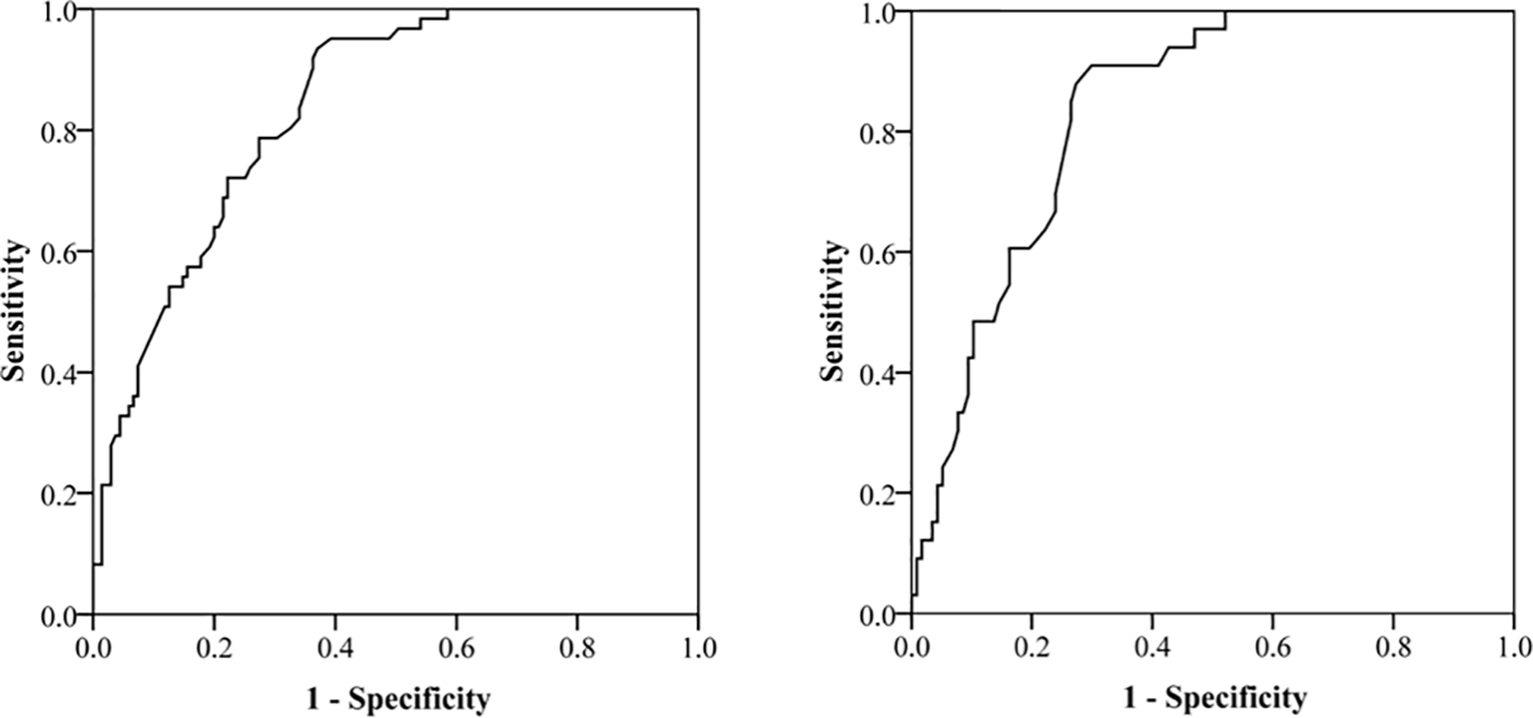
Receiver operating characteristic (ROC) curve for evaluating the performance of nomograms in evaluating aggressive behavior in pancreatic neuroendocrine tumors (PNET). The area under the curve is 0.84 (95% CI, 0.78–0.89) for total population and 0.84 (95% CI, 0.78–0.89) for patients with G1/G2 PNETs.

Subsequently, we evaluated the performance of above two nomograms in evaluating aggressive behavior in PNETs. The area under the curve (AUC) was 0.84 (95% CI, 0.77–0.90) for total population and was 0.84 (95% CI, 0.78–0.89) for patients with G1/G2 PNETs, respectively (**Figure 3**).

## Discussion

Aggressive behaviors may lead to a poor prognosis for PNET patients (8, 17, 18). It is important to recognize the relationship between clinical or biological features and aggressive behaviors. Previous studies have demonstrated that diabetes may be associated with metastasis of PNET (4). Another study showed that macroscopic morphology might help to estimate the malignancy of PNET (3). In the present study we found that neural invasion was an independent associated factor for aggressive behaviors in PNET both in total tumors and G1/G2 tumors. In addition, we developed two nomograms to predict aggressive behaviors in PNET. Those two models showed acceptable predictive performance.

Since originally described by Ernst in chondrosarcoma, neural invasion has been recognized as an important factor in the spread of carcinoma (5). Tsutsumi et al. indicated that neural invasion was an independent risk factor for recurrence of PNET, and they considered that PNEN cells infiltrated along the pancreatic neural plexus like pancreatic cancer leading to neural invasion (9). Kim et al. (19) showed that neural invasion was an independent prognostic factor of disease-free survival in PNET. Studies had also shown that neural invasion was associated with lymph node metastasis in pancreatic cancer (5). The neural infiltration in pancreatic cancer is an indicator of aggressive behaviors (6). Many studies have showed the risk factors of aggressive behavior in PNET (4, 10, 11, 20, 21). Woog et al. (22) showed that neural invasion was more common in tumors with lymph node metastasis. But to the best of our knowledge, few studies analyzed the association between neural invasion and aggressive behavior of PNET. Univariable and multivariable analyses both showed that tumors with higher grade, neural invasion and larger size were more prone to having aggressive behaviors. Fan et al. (4) reported that patients with diabetes might have a higher risk of neural invasion and distant metastasis. Accordingly, we further adjusted the data with diabetes. Interestingly, similar results were found in total population and G1/G2 or G3 patients. G3 PNET is divided into two subtypes, well-differentiated PNET and PNEC. Was nerve invasion more common in PNEC? Further researches are needed. Our study is a retrospective design. We cannot identify those well-differentiated PNET from G3 PNET.

The mechanism of how neural invasion affects the aggressive behaviors is still unknown. The effects of neural invasion on aggressive behaviors in pancreatic cancer may be associated with Schwann cells through cell-surface proteins (23). Schwann cells express myelin-associated glycoprotein (MAG) which can bind with mucin 1(MUC1) (6). MUC1-MAG signaling pathway contributes to the increased invasiveness and proliferation of pancreatic cancer cells (6). However, the reasons for the link between neural invasion and aggressive behaviors in PNET need further exploration. Moreover, targeting neural invasion maybe a potentially attractive therapeutic approach for patients with pancreatic ductal adenocarcinomas (6). Similarly, we speculated that targeting neural invasion may be also a therapeutical approach for patients with PNET.

There are also some limitations in this study. First, this study is a retrospective study and therefore has the general weaknesses of this type of study, such as selection bias. Second, the sample size was relatively small because PNET is a rare tumor. Third, the mechanism of neural invasion in PNET is still unclarified which should be analyzed in future studies. In addition, this study lacks survival data that will be helpful in predicting the prognosis of PNET. EPE and DM are the two major manifestations of aggressive behavior in the PNETs (11). In this study, EPE mainly included vascular invasion, organ invasion and lymph node metastases. Distal metastasis was not considered in our study because those patients were usually under biopsy examinations. We cannot evaluate the status of neural invasion. Finally, we did not perform an independent external validation because of the rare occurrence of PNET. However, we showed the data in total PNET and G1/G2 PNET and similar results were found. Calibration curve also showed acceptable performance for nomogam.

In conclusion, our study is the first one to report an association between neural invasion and aggressive behavior of PNET. This study has identified neural invasion may be a risk factor of aggressive behavior in patients with PNET. Our data also show that nomograms based on tumor size, tumor grade, and neural invasion can predict aggressive behavior in PNET with good performance.

## Data Availability Statement

The raw data supporting the conclusions of this article will be made available by the authors, without undue reservation.

## Ethics Statement

The studies involving human participants were reviewed and approved by the institutional review board of the First Affiliated Hospital, Zhejiang University School of Medicine. Written informed consent for participation was not required for this study in accordance with the national legislation and the institutional requirements.

## Author Contributions

HZ, XC, and ZW participated in the design of the study and wrote the manuscript. YW, HZ, XL, and WC analyzed the data. HZ, XC, and CG performed the experiments. All authors contributed to the article and approved the submitted version.

## Funding

Peak academic talent training fund of Jiangsu Province Hospital of Chinese Medicine (y2018rc04); Science and Technology Development Plan fund of Chinese Medicine of Jiangsu Province (ZD201907).

## Conflict of Interest

The authors declare that the research was conducted in the absence of any commercial or financial relationships that could be construed as a potential conflict of interest.

The handling editor declared a shared affiliation with one of the authors CG at the time of review.
